# Reorganized Brain Functional Network Topology in Presbycusis

**DOI:** 10.3389/fnagi.2022.905487

**Published:** 2022-05-26

**Authors:** Bing Guan, Yixi Xu, Yu-Chen Chen, Chunhua Xing, Li Xu, Song'an Shang, Jin-Jing Xu, Yuanqing Wu, Qi Yan

**Affiliations:** ^1^Department of Otolaryngology, Head and Neck Surgery, Clinical Medical College, Yangzhou University, Yangzhou, China; ^2^Department of Radiology, Nanjing First Hospital, Nanjing Medical University, Nanjing, China; ^3^Department of Otolaryngology, Nanjing First Hospital, Nanjing Medical University, Nanjing, China

**Keywords:** presbycusis, resting-state functional magnetic resonance imaging, graph theory, functional network, topological properties

## Abstract

**Purpose:**

Presbycusis is characterized by bilateral sensorineural hearing loss at high frequencies and is often accompanied by cognitive decline. This study aimed to identify the topological reorganization of brain functional network in presbycusis with/without cognitive decline by using graph theory analysis approaches based on resting-state functional magnetic resonance imaging (rs-fMRI).

**Methods:**

Resting-state fMRI scans were obtained from 30 presbycusis patients with cognitive decline, 30 presbycusis patients without cognitive decline, and 50 age-, sex-, and education-matched healthy controls. Graph theory was applied to analyze the topological properties of brain functional networks including global and nodal metrics, modularity, and rich-club organization.

**Results:**

At the global level, the brain functional networks of all participants were found to possess small-world properties. Also, significant group differences in global network metrics were observed among the three groups such as clustering coefficient, characteristic path length, normalized characteristic path length, and small-worldness. At the nodal level, several nodes with abnormal betweenness centrality, degree centrality, nodal efficiency, and nodal local efficiency were detected in presbycusis patients with/without cognitive decline. Changes in intra-modular connections in frontal lobe module and inter-modular connections in prefrontal subcortical lobe module were found in presbycusis patients exposed to modularity analysis. Rich-club nodes were reorganized in presbycusis patients, while the connections among them had no significant group differences.

**Conclusion:**

Presbycusis patients exhibited topological reorganization of the whole-brain functional network, and presbycusis patients with cognitive decline showed more obvious changes in these topological properties than those without cognitive decline. Abnormal changes of these properties in presbycusis patients may compensate for cognitive impairment by mobilizing additional neural resources.

## Introduction

Presbycusis, defined as progressive bilateral sensorineural high-frequency hearing loss, has become a pervasive public health issue frequently accompanied with social isolation, communication, language, and speech processing problems (Gates and Mills, 2005; Dubno et al., 2008; Panza et al., 2015). Approximately 35–50% of adults aged 65 years or older suffered from presbycusis with the aging of the global population, consequently bringing a great social and economic burden (Rutherford et al., 2018). The main clinical features of presbycusis are characterized by slow central processing of auditory information, impaired localization of sound sources, and reduced ability to distinguish speech in noisy environments (Gates and Mills, 2005). In addition, presbycusis has been independently associated with cognitive decline and increased risk of dementia (Ford et al., 2018; Loughrey et al., 2018). This has been a serious negative impact on the daily life and social interaction of the elderly population.

Although the loss of sensory hair cell and neurons in the cochlea is the main cause of presbycusis, there is growing awareness that presbycusis is also associated with the structural and functional changes in the central auditory pathway and other regions in the central nervous system (Kazee et al., 1995; Ouda et al., 2015). Besides, less is known about its association with cognitive impairment. Several neuroimaging studies of presbycusis have demonstrated a variety of brain functional changes outside the auditory pathway. For example, Chen et al. (2018b, 2020) found that age-related hearing loss was associated with a decline in spontaneous activity of the auditory cortex, whereas impaired cognitive/executive function was associated with increased spontaneous activity in the prefrontal cortex; the decline of cognitive function in presbycusis patients may be closely related to the functional changes of the default mode network (DMN). Xing et al. proposed that the disorder of brain functional network architecture in the frontal lobe plays a crucial role in the executive dysfunction of presbycusis, and early biomarkers of FC alteration may exist before distinct cognitive impairment is detected in patients with presbycusis (Xing et al., 2020, 2021a,b).

Resting-state functional MRI (rs-fMRI) can non-invasively measure spontaneous activity in the human brain (Biswal et al., 1995). It mainly uses the blood-oxygenation level-dependent (BOLD) signal as a neurophysiological index and has been successfully applied to various clinical diseases such as mitochondrial encephalomyopathy with lactic acidosis and stroke-like episodes (MELAS) (Wang et al., 2020), type 2 diabetes mellitus (Xu et al., 2019), and obstructive sleep apnea (Chen et al., 2018a). Graph theory studies the complex network of the human brain connectome by measuring the topological properties of the region of interest (ROI) or that network associated with a particular function throughout the brain (Bullmore and Sporns, 2009; Smitha et al., 2017). The rs-fMRI brain network analysis method based on graph theory has shown that the human brain functional networks have many important topological properties, such as small-world properties, modular structure, and rich-club organization (Wang et al., 2020), and it can explore the functional connections between the whole brain and the local brain regions. Several studies have used graph theory analysis to reveal the destroyed topological properties of the functional network in several neurological diseases (Shi et al., 2021; You et al., 2021; Lan et al., 2022), playing a role in monitoring the disease status and providing a novel insight regarding the neurobiological mechanisms in these patients. As well, Xu et al. applied the graph theory analysis to sudden sensorineural hearing loss and found that nodal betweenness of the limbic network increased, which may indicate a plastic reorganization procedure of the brain to compensate for the hearing loss and cognitive decline (Xu et al., 2016). However, how the topology of brain functional network changes in the presbycusis patients has not been thoroughly studied yet.

In this study, we employed rs-fMRI to construct brain functional networks of presbycusis patients with or without cognitive decline and the healthy controls (HCs) and compared the diversity of entire brain functional network topology properties among the three groups using graph theory analysis. In this study, we hypothesized that: (1) presbycusis patients, especially those with cognitive decline, have topological disturbance and reorganization in the whole brain, (2) hearing loss and cognitive decline may be associated with functional network property alteration in the presbycusis patients with cognitive decline, and (3) there may be some alterations in topological properties to make a compensation to lessen the consequences caused by hearing loss and cognitive decline.

## Materials and Methods

### Subjects

All the subjects provided written informed consent before their participation in the study protocol, and 110 participants (all right handed and educated for at least 8 years) were enrolled in this study, which included 60 presbycusis patients recruited from the otolaryngology department and 50 age-, gender-, and education-matched HCs recruited through community health census or online advertising. Hearing loss was assessed by the speech-frequency pure tone average (PTA) of thresholds at the frequencies of 0.25, 0.5, 1, 2, 4, and 8 kHz in the better hearing ears. The PTA value of 25 dB was accepted as the normal hearing threshold limit. Inclusion criteria of the presbycusis were average PTA > 25 dB in the better hearing ear and age ≥ 60 years. Tympanometry was performed with a Madsen Electronics Zodiac 901 Middle Ear Analyzer (GN Otometrics) to confirm normal middle-ear function. Approval for the study was obtained from the Research Ethics Committee of Nanjing Medical University.

Exclusion criteria included the following: (1) ear diseases that affected hearing threshold, including tinnitus, hyperacusis (Khalfa et al., 2002), and Meniere's disease (Lopez-Escamez et al., 2015); (2) a history of ototoxic drug therapy, otologic surgery, noise exposure, or hearing aid use; (3) conductive hearing loss (a mean air-bone difference at 0.5, 1, 2, and 4 kHz) > 10 dB in one or both ears; and (4) severe smoking, alcohol abuse, brain damage, stroke, Alzheimer's disease, Parkinson's disease, major depression, epilepsy, mental or neurological disorders, and major diseases (such as anemia, thyroid dysfunction, and cancer); and (5) MRI contraindications.

### Neuropsychological Assessment

A comprehensive test of cognitive status was performed on all participants using Montreal Cognitive Assessment (MoCA), including 8 cognitive domains, namely, visual space and executive function, attention, memory, naming, abstract thinking, language, delayed recall, and orientation. The test was carried out in a quiet environment and all the subjects were expected to be relaxed and conscious. MoCA is commonly used to screen for Mild Cognitive Impairment (MCI), which has high sensitivity (Hobson, 2015). The MoCA test result has a total score of 30 points, with a final score ≥ 26 being considered normal. According to the MoCA score, the presbycusis patients were divided into 30 patients with cognitive decline and 30 without cognitive decline.

### MRI Acquisition

Subjects were scanned under resting conditions using a 3.0 Tesla MRI scanner (Ingenia, Philips Medical Systems, Netherlands) with an 8-channel receiver array head coil. During scanning, the subjects were supposed to lie quietly with their eyes closed and avoid head movement during the scan, but not to fall asleep or think about anything special. To reduce head motion and scanner noise, foam pad and earplugs were used. According to the manufacturer's specifications, the earplugs (Hearos Ultimate Softness Series, USA) could attenuate scanner noise by almost 32 dB. Resting-state functional images were obtained axially using a gradient echo-planar imaging sequence as follows: repetition time (TR) = 2,000 ms, echo time (TE) = 30 ms, slices = 36, thickness = 4 mm, gap = 0 mm, field of view (FOV) = 240 × 240 mm, acquisition matrix = 64 × 64, and flip angle (FA) = 90°. The voxel size was 3.75 × 3.75 × 4.0 mm^3^. Structural images were obtained using a three-dimensional turbo fast echo (3D-TFE) T1WI sequence and following scan parameters: TR/TE = 8.1/3.7 ms, slices = 170, thickness = 1 mm, gap = 0 mm, FA = 8°, acquisition matrix = 256 × 256, and FOV = 256 × 256 mm. The functional sequence lasted for 8 min and 8 s, and the structural sequence lasted for 5 min and 29 s.

### Functional Imaging Data Preprocessing

The rs-fMRI images were preprocessed using GRETNA (a graph theoretical network analysis toolbox for imaging connectomics) (Wang et al., 2015) (2.0.0A http://www.nitrc.org/projects/gretna/). The preprocess is as follows. (1) The first 10 volumes were removed because of the possible disequilibrium of the initial magnetization and subject's environmental adaptation. (2) Slice-timing correction and realignment were performed for the remaining 220 images. Any subjects with a head motion > 2.0 mm translation or a 2.0° rotation in any direction were excluded from analysis. (3) The remaining data were spatially normalized to the Montreal Neurological Institute template (resampling voxel size = 3 × 3 × 3 mm^3^). (4) Subsequently, several nuisance signals were regressed from the data including head motion, the global mean, and signals from white matter and the cerebrospinal fluid. (5) At the end of preprocessing, the time series of each voxel was temporally bandpass filtered (0.01–0.08 Hz) and linearly detrended.

### Functional Connectivity Matrix and Graph Construction

The functional network was constructed using GRETNA (Wang et al., 2015), and the results were visualized using BrainNet Viewer (Xia et al., 2013). A network is composed of many nodes and the connecting edges between these nodes. Nodes represent brain regions, and an edge occurs when there is an anatomical connection or functional correlation between two nodes. In this study, we constructed the brain functional network for each subject according to the automated anatomical labeling atlas with 90 brain regions of interest (AAL90) template, which consists of 90 regions of interest (ROIs) (Tzourio-Mazoyer et al., 2002).

Each region was taken as a network node, and then, the mean time series was obtained for each region; the partial correlations of the mean time series between all pairs of the nodes (representing their conditional dependences by excluding the effects of the other 88 regions) were regarded as the edges of the network. Then, a partial correlation matrix (90 × 90) was generated for each subject and converted to an undirected binary matrix according to a predefined threshold (aij = 1, if the absolute partial correlation between regions i and area j exceeded the threshold, otherwise aij = 0). The networks of individual subjects were different in the number of edges; a range of sparse thresholds S, defined as the fraction of the total number of edges remaining in the network (Achard and Bullmore, 2007), was applied to the correlation matrices so that each graph had the same number of edges; its minimum value was set so that the average node degree of the threshold network was 2log(N), where N was the number of nodes (Fornito et al., 2010). The threshold range generated by this process was 0.06 S 0.4, and the interval was 0.01. The resulting 35 binarized connectivity matrices for each subject could estimate the sparse properties of small-worldness and the smallest possible number of false edges (Watts and Strogatz, 1998). For the brain networks at each sparsity level, we calculated both the global and node network metrics.

### Network Analysis

#### Global and Nodal Metrics

By calculating the global network parameters and regional node parameters, the global topological structure of the brain functional network and the regional attributes of each node were represented for the brain networks at each sparsity threshold (Rubinov and Sporns, 2010; Wang et al., 2010). The global network metrics are composed of (1) small-world properties including clustering coefficient (Cp), characteristic path length (Lp), normalized clustering coefficient (γ), normalized characteristic path length (λ), and small-worldness (σ) and (2) network efficiency parameters including global network efficiency (Eglob) and local network efficiency (Eloc). γ = Cp/Cprand, λ = Lp/Lprand, Cprand, and Lprand were calculated from a random network.

High Cp, γ, and Eloc can show functional segregation of the brain network, which indicates the local interconnectivity of a network. Low Lp, λ, and high Eglob can reflect the functional integration in the brain, which is the ability for global information communication. Small-worldness quantifies the balance between integration/global processing (low characteristic path length) and segregation/local processing (high mean clustering coefficient). A small-world network typically has both the high mean clustering coefficient typical of regular lattice networks (γ > 1) and the small characteristic path length typical of random networks (λ ≈ 1) (Watts and Strogatz, 1998).

To explore nodal properties of the brain functional network, we calculated the betweenness centrality (BC), degree centrality (DC), nodal efficiency (NE), and nodal local efficiency (NLE). Betweenness centrality is a measure of a node's influence on the overall flow of information in the graph. Greater betweenness centrality means that most information flows through the node (Linton, 1978). Degree centrality is a measure of the number of direct connections with other nodes in the graph. Greater degree centrality means more connections (Rubinov and Sporns, 2010). Nodal efficiency represents the node efficiency of a given node, indicating the efficiency of the parallel information transmission of the node in the network (Rubinov and Sporns, 2010). Nodal local efficiency, defined as the inverse of the shortest average path length in a subgraph comprising of node and its adjacent neighbors, is considered a measure of fault tolerance in a network as it characterizes how well-information is exchanged by neighbors if the node is removed (Achard and Bullmore, 2007). Because area under the curve (AUC) can select single threshold calculation independently, and is highly sensitive about topology structure of brain disease abnormally, we calculated the AUC for each network metric (Zhang et al., 2011).

#### Modular Architecture

The AAL90 template divided the 90 ROIs into six sub-modules, namely, frontal lobe module, prefrontal lobe module, subcortical module, temporal lobe module, occipital lobe module, and parietal lobe module. We calculated the mean strength of intra- and inter-modular connections among the presbycusis patients with cognitive decline, the presbycusis patients without cognitive decline, and the HCs. For each subject, the mean strength of the intra-module was the average number of intra-modular connections of the selected module, and the mean strength of the inter-module was the average number of inter-modular connections between the selected module and other modules.

#### Rich-Club Organization

The rich-club organization is present in the brain network when the high-degree nodes are more massively interconnected than that expected by chance (Van Den Heuvel and Sporns, 2011). Rich-club nodes were chosen as the top 10 (12%) brain regions with the highest average nodal degree on the basis of the group-average cortical network (Van Leijsen et al., 2019). Based on the categorization of the nodes into “rich-club” nodes and “non-rich-club” nodes, the edges of the functional network were classified into three connection classes: “rich-club connections,” linking two rich-club nodes; “feeder connections,” linking one rich-club node to one non-rich-club node; and “local connections,” linking two non-rich-club nodes (Sporns, 2011; Van Den Heuvel and Sporns, 2011).

### Statistical Analysis

Statistical analyses were performed using SPSS 21.0 (SPSS, Inc., Chicago, IL, USA). Categorical variables were investigated with a chi-squared test. The normality of distribution was assessed using the Shapiro-Wilk test. Non-parametric tests were applied if the data were identified as not normally distributed, while normally distributed continuous variables were investigated with a one-way ANOVA test for three groups. *p* < 0.05 was statistically significant. The AUCs of all the network metrics of the presbycusis patients with cognitive decline, the presbycusis patients without cognitive decline, and the HCs were separately statistically analyzed using a one-way ANOVA or nonparametric tests. Then, two-sample *t*-tests were performed as *post-hoc* tests between any two groups if the ANOVA test or nonparametric test showed significant differences. Bonferroni correction for multiple comparisons was carried out for nodal analysis to diminish the Type I error of simple effects (*p* < 0.05). Age, gender, and education were treated as covariates in all the statistical analyses.

## Results

### Demographics and Clinical Data

The demographics and clinical characteristics of the three groups are summarized in Table 1. There were no significant differences between the presbycusis group and HCs in terms of age, sex, and education level. Besides, no significant difference was revealed in PTA between the left and right ears of the presbycusis patients and the HCs. For cognitive assessment, 30 patients with presbycusis performed significantly poorer in MoCA scores than other groups (*p* < 0.001).

**Table 1:** Demographics of the presbycusis patients and HCs.

	**PCD (*n* = 30)**	**PNCD (*n* = 30)**	**HCs (*n* = 50)**	***P*-value**
Age (year)	63.03 ± 7.30	62.47 ± 7.12	61.08 ± 3.93	0.321^a^
Sex (M/F)	14/16	12/18	24/26	0.776^b^
Education (years)	10.40 ± 2.18	11.47 ± 1.72	10.78 ± 1.79	0.087^a^
PTA of left ear (dB HL)	33.08 ± 3.79	33.11 ± 5.00	17.93 ± 5.84	<0.001^*^^a^
PTA of right ear (dB HL)	33.16 ± 6.66	33.52 ± 5.28	17.40 ± 5.19	<0.001^*^^a^
Average PTA of both ears (dB HL)	33.12 ± 3.60	33.32 ± 4.14	17.67 ± 5.12	<0.001^*^^a^
MoCA scores	24.57 ± 0.77	26.63 ± 0.72	26.74 ± 1.23	<0.001^*^^a^

*Data are represented as Mean ± SD*.

* *p <0.001*.

a *The p-values are obtained by using one-way ANOVA*.

b *The p-values are obtained by using χ^2^-test. M, male; F, female; PTA, puretone audiometry; PCD, presbycusis with cognitive decline; PNCD, presbycusis without cognitive decline; HCs, healthy controls*.

### Group Differences in Global Network Organization

**Figure 1:**
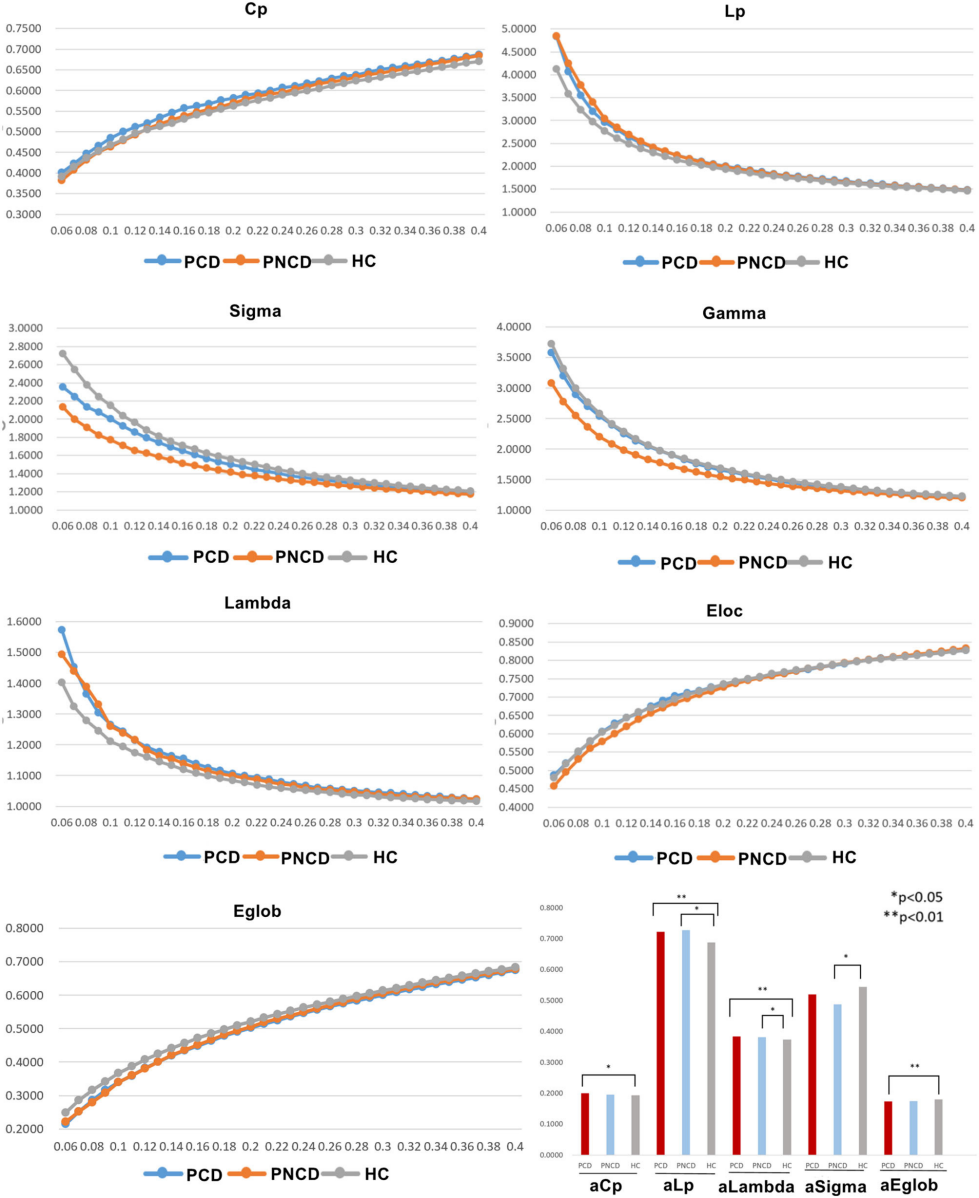
The differences in global metrics of the brain functional networks among presbycusis patients with cognitive decline, presbycusis patients without cognitive decline, and healthy controls. PCD, presbycusis patients with cognitive decline; PNCD, presbycusis patients without cognitive decline; HC, healthy controls; Cp, clustering coefficient; Lp, shortest path length; sigma (δ), small-world characteristic; gamma (γ), normalized clustering coefficient; lambda (λ), normalized characteristic path length; Eloc, local efficiency; Eglob, global efficiency; aCp, AUC in Cp; aLp, AUC in Lp; aLambda, AUC in Lambda; aEglob, AUC in Eglob. *p < 0.05, **p < 0.01.

In the given threshold range, all presbycusis patients with cognitive decline, presbycusis patients without cognitive decline, and HCs exhibited a typical small-worldness (γ > 1, λ ≈ 1, σ > 1). Cp, Eloc, and Eglob values of the three groups increased with increasing threshold, while Lp, γ, λ, and σ values decreased. Among these three groups, significant group differences in global network metrics (Cp *p* = 0.043, Lp *p* = 0.001, λ *p* < 0.001, σ *p* = 0.038, Eglob *p* = 0.004) were observed. Compared with the HCs, the presbycusis patients with cognitive decline showed significantly increased values in Cp (*p* = 0.037), Lp (*p* = 0.002), and λ (*p* < 0.001), and significantly decreased values in Eglob (*p* = 0.005). The presbycusis patients without cognitive decline showed significantly increased values in Lp (*p* = 0.031) and λ (*p* = 0.046) and significantly decreased values in σ (*p* = 0.033). The presbycusis patients with cognitive decline and without cognitive decline did not differ significantly in the global network metrics. In addition, there were no significant difference among the three groups in γ (*p* = 0.068) and Eloc (*p* = 0.449) (Figure 1).

### Group Differences in Nodal Network Metrics

**Figure 2:**
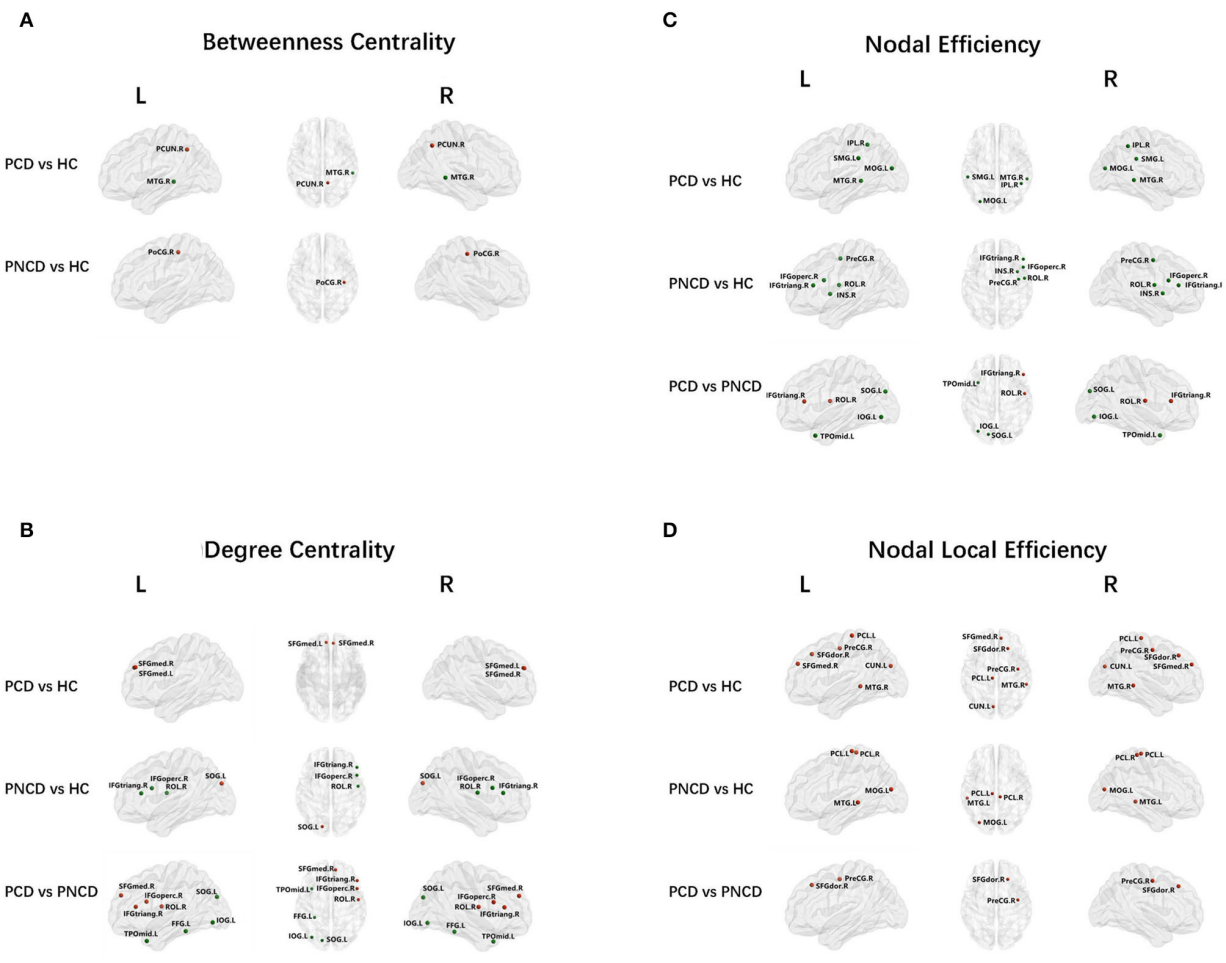
Nodal network analysis for presbycusis patients with cognitive decline, patients with no cognitive decline, and healthy controls. Nodes showing increased (red) and decreased (green) betweenness centrality **(A)** in brain functional networks for the comparisons of PCD vs. HC and PNCD vs. HC. Nodes showing increased (red) and decreased (green) degree centrality **(B)**, nodal efficiency **(C)**, and nodal local efficiency **(D)** in brain functional networks for the comparisons of PCD vs. HC, PNCD vs. HC, and PCD vs. PNCD. Correction for multiple comparisons was performed using the Bonferroni calibration (*p* < 0.05). PCD, presbycusis patients with cognitive decline; PNCD, presbycusis patients without cognitive decline; HC, healthy controls.

As shown in Figure 2A, Table 2 (*p* < 0.05, Bonferroni corrected), for nodal metrics, compared with the HCs, the presbycusis patients with cognitive decline showed increased BC in the right precuneus (PCUN.R) and decreased BC in the right middle temporal gyrus (MTG.R), while the presbycusis patients without cognitive decline showed increased BC in the right postcentral gyrus (PoCG.R).

**Table 2:** Nodes with significant grouping differences in nodal indicators.

	**X (mm)**	**y (mm)**	**Z (mm)**		***P*-value**
**PCD vs.HCs (Bc)**					
Precuneus_R	9.98	−56.05	43.77	PCD>HC	0.008
Temporal_Mid_R	57.47	−37.23	−1.47	PCD < HC	0.017
**PNCD vs.HCs (Bc)**					
Postcentral_R	41.43	−25.49	52.55	PNCD>HC	0.040
**PCD vs.HCs (Dc)**					
Frontal_Sup_Medial_L	−4.80	49.17	30.89	PCD>HC	0.021
Frontal_Sup_Medial_R	9.10	50.84	30.22	PCD>HC	0.016
**PNCD vs. HCs (Dc)**					
Frontal_Inf_Oper_R	50.20	14.98	21.41	PNCD < HC	0.003
Frontal_Inf_Tri_R	50.33	30.16	14.17	PNCD < HC	0.009
Rolandic_Oper_R	52.65	−6.25	14.63	PNCD < HC	0.025
Occipital_Sup_L	−16.54	−84.26	28.17	PNCD>HC	0.028
**PCD vs.PNCD (Dc)**					
Frontal_Inf_Oper_R	50.20	14.98	21.41	PCD>PNCD	0.026
Frontal_Inf_Tri_R	50.33	30.16	14.17	PCD>PNCD	0.011
Rolandic_Oper_R	52.65	−6.25	14.63	PCD>PNCD	0.032
Frontal_Sup_Medial_R	9.10	50.84	30.22	PCD>PNCD	0.040
Occipital_Sup_L	−16.54	−84.26	28.17	PCD < PNCD	0.021
Occipital_Inf_L	−36.36	−78.29	−7.84	PCD < PNCD	0.013
Fusiform_L	−31.16	−40.30	−20.23	PCD < PNCD	0.036
Temporal_Pole_Mid_L	−36.32	14.59	−34.08	PCD < PNCD	0.029
**PCD vs.HCs (Ne)**					
Occipital_Mid_L	−32.39	−80.73	16.11	PCD < HC	0.017
Parietal_Inf_R	46.46	−46.29	49.54	PCD < HC	0.041
SupraMarginal_L	−55.79	−33.64	30.45	PCD < HC	0.043
Temporal_Mid_R	57.47	−37.23	−1.47	PCD < HC	0.024
**PNCD vs.HCs (Ne)**					
Precentral_R	41.37	−8.21	52.09	PNCD < HC	0.012
Frontal_Inf_Oper_R	50.20	14.98	21.41	PNCD < HC	0.002
Frontal_Inf_Tri_R	50.33	30.16	14.17	PNCD < HC	0.007
Rolandic_Oper_R	52.65	−6.25	14.63	PNCD < HC	0.013
Insula_R	39.02	6.25	2.08	PNCD < HC	0.028
**PCD vs.PNCD (Ne)**					
Frontal_Inf_Tri_R	50.33	30.16	14.17	PCD>PNCD	0.019
Rolandic_Oper_R	52.65	−6.25	14.63	PCD>PNCD	0.043
Occipital_Sup_L	−16.54	−84.26	28.17	PCD < PNCD	0.007
Occipital_Inf_L	−36.36	−78.29	−7.84	PCD < PNCD	0.015
Temporal_Pole_Mid_L	−36.32	14.59	−34.08	PCD < PNCD	0.026
**PCD vs.HCs (NLe)**					
Precentral_R	41.37	−8.21	52.09	PCD>HC	0.032
Frontal_Sup_R	21.90	31.12	43.82	PCD>HC	0.016
Frontal_Sup_Medial_R	9.10	50.84	30.22	PCD>HC	0.003
Cuneus_L	−5.93	−80.13	27.22	PCD>HC	0.010
Paracentral_Lobule_L	−7.63	−25.36	70.07	PCD>HC	0.024
Temporal_Mid_R	57.47	−37.23	−1.47	PCD>HC	0.004
**PNCD vs.HCs (NLe)**					
Occipital_Mid_L	−32.39	−80.73	16.11	PNCD>HC	0.014
Paracentral_Lobule_L	−7.63	−25.36	70.07	PNCD>HC	0.004
Paracentral_Lobule_R	7.48	−31.59	68.09	PNCD>HC	0.009
Temporal_Mid_L	−55.52	−33.80	−2.20	PNCD>HC	0.005
**PCD vs.PNCD (NLe)**					
Precentral_R	41.37	−8.21	52.09	PCD>PNCD	0.042
Frontal_Sup_R	21.90	31.12	43.82	PCD>PNCD	0.038

*PCD, presbycusis with cognitive decline; PNCD, presbycusis without cognitive decline; HCs, healthy controls*.

For the presbycusis patients with cognitive decline, there was a significantly increased DC in bilateral superior frontal gyrus, medial (SFGmed.L and SFGmed.R) compared with the HCs. For the presbycusis patients without cognitive decline, there was a significantly decreased DC in the right inferior frontal gyrus, opercular part (IFGoperc.R), the right inferior frontal gyrus, triangular part (IFGtriang.R), and the right rolandic operculum (ROL.R) but an increased DC in the left superior occipital gyrus (SOG.L) compared with the HCs. Additionally, a significantly higher DC was found in the right inferior frontal gyrus, opercular part (IFGoperc.R), the right inferior frontal gyrus, triangular part (IFGtriang.R), the right rolandic operculum (ROL.R), and the right superior frontal gyrus, medial (SFGmed.R), but a significantly lower DC was found in the left superior occipital gyrus (SOG.L), the left inferior occipital gyrus (IOG.L), the left fusiform gyrus (FFG.L), and the left temporal pole: middle temporal gyrus (TPOmid.L) for the presbycusis patients with cognitive decline compared with the presbycusis patients without cognitive decline (Figure 2B).

Compared with the HCs, the presbycusis patients with cognitive decline showed a decreased NE in the left middle occipital gyrus (MOG.L), the right inferior parietal, supramarginal and angular gyri (IPL.R), the left supramarginal gyrus (SMG.L), and the right middle temporal gyrus (MTG.R), while the presbycusis patients without cognitive decline showed a decreased NE in the right precental gyrus (PreCG.R), the right inferior frontal gyrus, opercular part (IFGoperc.R), the right inferior frontal gyrus, triangular part (IFGtriang.R), the right rolandic operculum (ROL.R), and the right insula (INS.R). When compared with the presbycusis patients without cognitive decline, the presbycusis patients with cognitive decline showed a significantly increased NE in the right inferior frontal gyrus, triangular part (IFGtriang.R), and the right rolandic operculum (ROL.R) but a significantly decreased NE in the left superior occipital gyrus (SOG.L), the left inferior occipital gyrus (IOG.L), and the left temporal pole: middle temporal gyrus (TPOmid.L) (Figure 2C).

A significantly increased NLE was observed in the right precentral gyrus (PreCG.R), the right superior frontal gyrus, dorsolateral (SFGdor.R), the right superior frontal gyrus, medial (SFGmed.R), the left cuneus (CUN.L), the left paracentral lobule (PCL.L), and the right middle temporal gyrus (MTG.R) for the presbycusis patients with cognitive decline compared with the HCs. For the presbycusis patients without cognitive decline, there was a significantly increased NLE in the left middle occipital gyrus (MOG.L), bilateral paracentral lobule (PCL.L and PCL.R), and the left middle temporal gyrus (MTG.L) compared with the HCs. Furthermore, a significantly higher NLE was found in the right precentral gyrus (PreCG.R) and the right superior frontal gyrus, dorsolateral (SFGdor.R) for the presbycusis patients with cognitive decline compared with the presbycusis patients without cognitive decline (Figure 2D).

### Group Differences in Intra- and Inter-modular Connections

**Figure 3:**
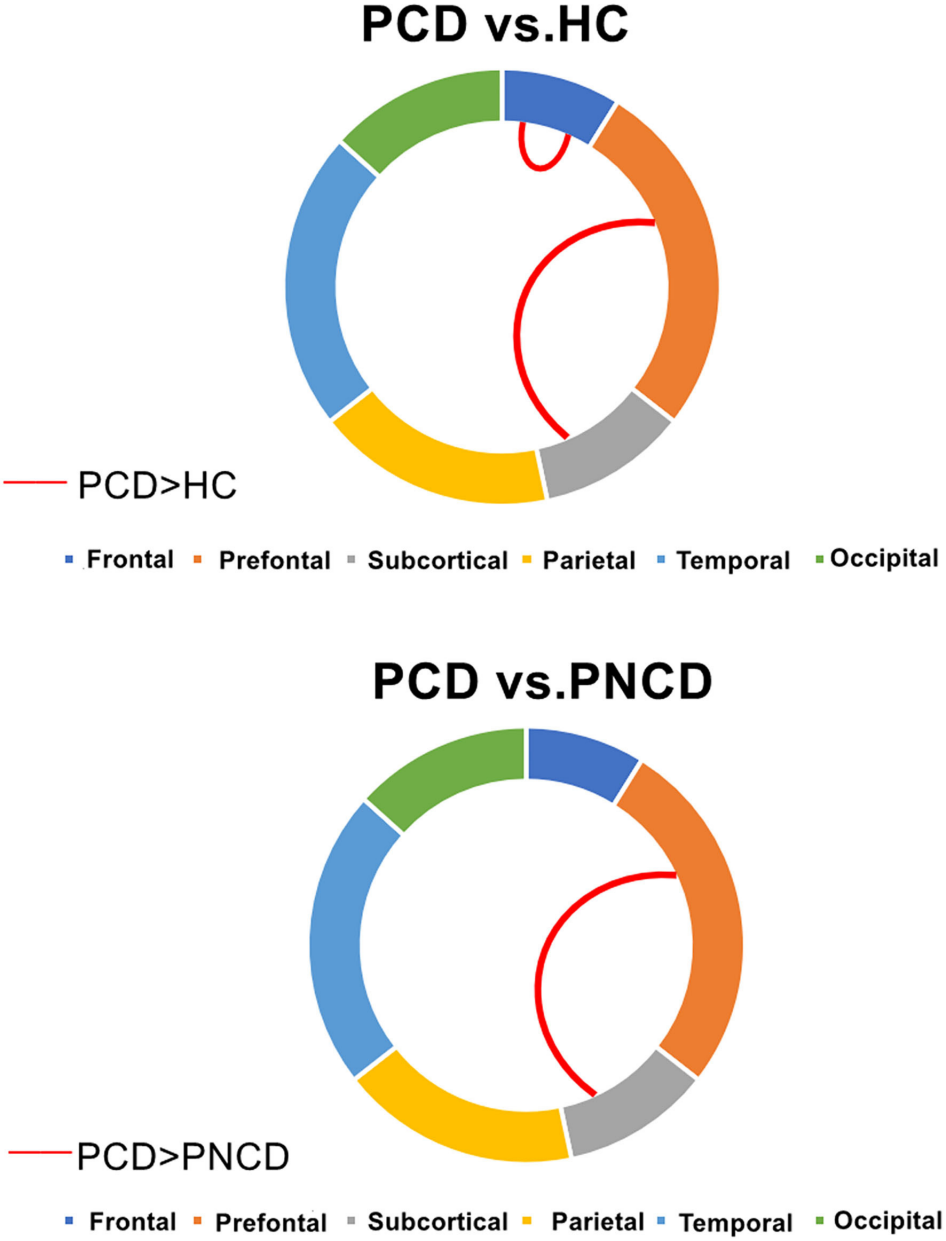
Presbycusis patients with cognitive decline, presbycusis patients without cognitive decline, and healthy controls have significant differences in intra- and inter-modular connections. PCD, presbycusis patients with cognitive decline; PNCD, presbycusis patients with no cognitive decline; HC, healthy controls. Red lines represented significantly more connections.

There were significant group differences in 1 case of intra-module connection and 1 case of inter-module connection. *Post-hoc* analysis revealed that when compared with the HCs, the presbycusis patients with cognitive decline showed increased connection strength in the frontal lobe module and prefrontal subcortical lobe module. When compared with the presbycusis patients without cognitive decline, the presbycusis patients with cognitive decline showed increased connection strength in the prefrontal subcortical lobe module (all *p* < 0.05 with Bonferroni correction) (Figure 3).

### Group Differences in Rich-Club Organization

**Figure 4:**
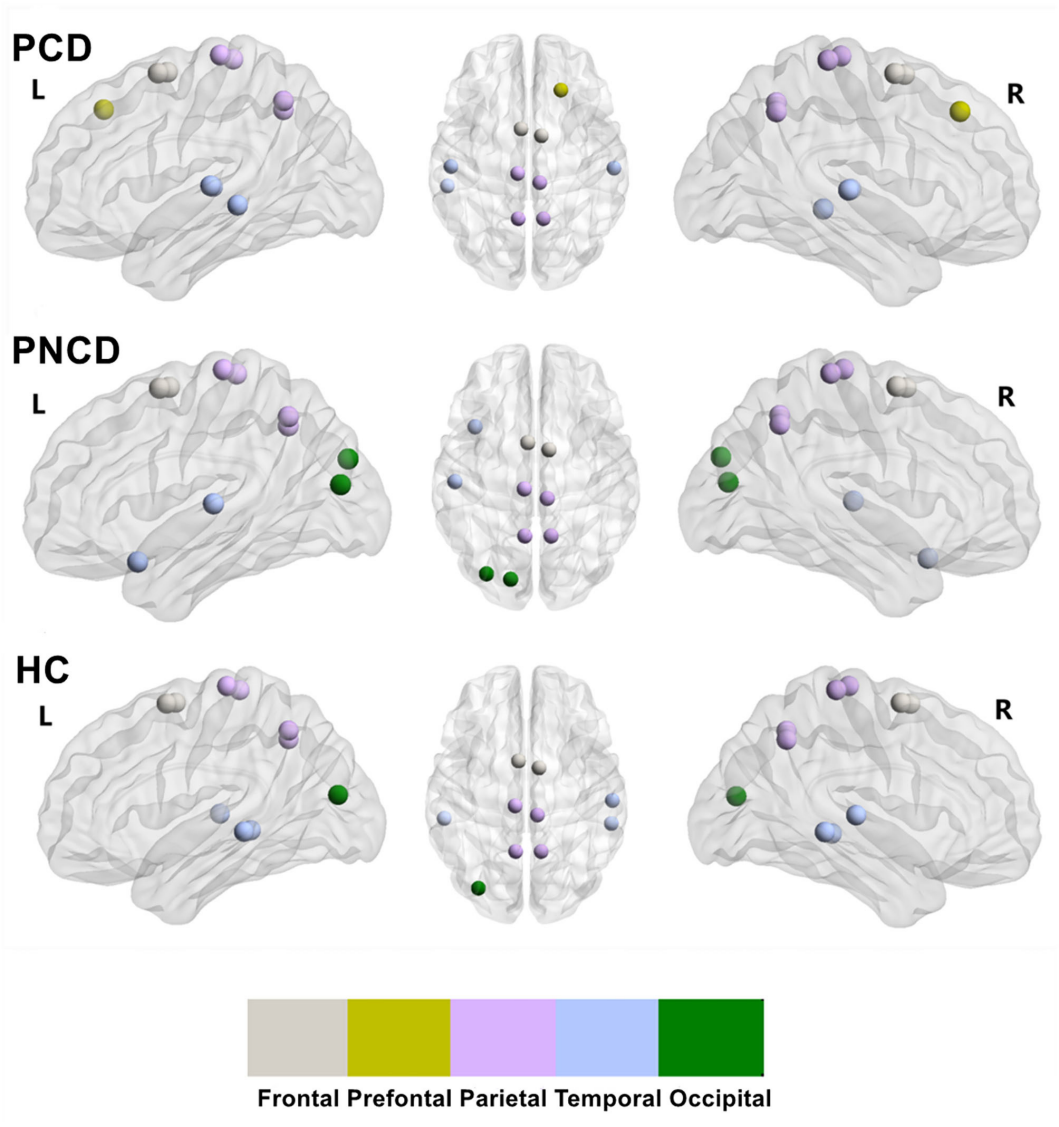
The rich-club organization for presbycusis patients with cognitive decline, presbycusis patients without cognitive decline, and healthy controls. PCD, presbycusis patients with cognitive decline; PNCD, presbycusis patients with no cognitive decline; HC, healthy controls. The distribution of rich-club nodes in the whole brain for each group.

Based on the group-averaged functional network, the rich-club nodes were defined as the top 10 (12%) brain regions with the highest average nodal degree of all regions for each group (Table 3). The distribution pattern of rich-club nodes was relatively similar between the presbycusis patients without cognitive decline and the HCs, but it was different from the presbycusis patients with cognitive decline (Figure 4). The rich-club nodes in the HCs mainly comprised the bilateral Supp_Motor_Area, Precuneus_L, bilateral Paracentral_Lobul, bilateral Temporal_Mid, Temporal_Sup_R, Precuneus_R, and Occipital_Mid_L. The newly formed rich-club nodes in the presbycusis patients without cognitive decline were mainly composed of the bilateral Supp_Motor_Area, bilateral Precuneus, bilateral Paracentral_Lobule, Occipital_Mid_L, Temporal_Pole_Sup_L, Occipital_Sup_L, and Temporal_Sup_L. Differently, the newly formed rich-club nodes in the presbycusis patients with cognitive decline were mainly composed of the bilateral Supp_Motor_Area, bilateral Precuneus, bilateral Temporal_Sup, Temporal_Mid_L, bilateral Paracentral_Lobule, and Frontal_Sup_R. However, no significant group differences were observed in the mean strength of feeder, local, and rich-club connections.

**Table 3:** Rich–club nodes for each group.

**Rich–club nodes for each group**					
	**X (mm)**	**Y (mm)**	**Z (mm)**	**Functional**	**Anatomical**
				**classification**	**classification**
**PCD**					
Supp_Motor_Area_R	8.62	0.17	61.85	Association	Frontal
Supp_Motor_Area_L	−5.32	4.85	61.38	Association	Frontal
Precuneus_L	−7.24	−56.07	48.01	Association	Parietal
Precuneus_R	9.98	−56.05	43.77	Association	Parietal
Temporal_Sup_R	58.15	−21.78	6.80	Association	Temporal
Temporal_Sup_L	−53.16	−20.68	7.13	Association	Temporal
Temporal_Mid_L	−55.52	−33.80	−2.20	Association	Temporal
Paracentral_Lobule_R	7.48	−31.59	68.09	Association	Parietal
Paracentral_Lobule_L	−7.63	−25.36	70.07	Association	Parietal
Frontal_Sup_R	21.90	31.12	43.82	Association	Prefrontal
**PNCD**					
Supp_Motor_Area_R	8.62	0.17	61.85	Association	Frontal
Supp_Motor_Area_L	−5.32	4.85	61.38	Association	Frontal
Precuneus_L	−7.24	−56.07	48.01	Association	Parietal
Paracentral_Lobule_R	7.48	−31.59	68.09	Association	Parietal
Precuneus_R	9.98	−56.05	43.77	Association	Parietal
Paracentral_Lobule_L	−7.63	−25.36	70.07	Association	Parietal
Occipital_Mid_L	−32.39	−80.73	16.11	Association	Occipital
Temporal_Pole_Sup_L	−39.88	15.14	−20.18	Paralimbic	Temporal
Occipital_Sup_L	−16.54	−84.26	28.17	Association	Occipital
Temporal_Sup_L	−53.16	−20.68	7.13	Association	Temporal
**HCs**					
Supp_Motor_Area_R	8.62	0.17	61.85	Association	Frontal
Supp_Motor_Area_L	−5.32	4.85	61.38	Association	Frontal
Precuneus_L	−7.24	−56.07	48.01	Association	Parietal
Paracentral_Lobule_R	7.48	−31.59	68.09	Association	Parietal
Temporal_Mid_R	57.47	−37.23	−1.47	Association	Temporal
Temporal_Mid_L	−55.52	−33.80	−2.20	Association	Temporal
Paracentral_Lobule_L	−7.63	−25.36	70.07	Association	Parietal
Temporal_Sup_R	58.15	−21.78	6.80	Association	Temporal
Precuneus_R	9.98	−56.05	43.77	Association	Parietal
Occipital_Mid_L	−32.39	−80.73	16.11	Association	Occipital

*PCD, presbycusis with cognitive decline; PNCD, presbycusis without cognitive decline; HCs, healthy controls*.

## Discussion

Based on graph theoretical analysis of rs-fMRI data, this study constructed a whole-brain functional network for presbycusis patients and HCs and detected the topological differences in the brain functional network of presbycusis patients. The main results were as follows: (1) All these three groups exhibited typical small-world topology. When presbycusis patients with/without cognitive decline were compared with the HCs, increased/decreased global metrics were found. (2) When compared with the HCs, abnormal nodal centralities were found in widespread brain regions in the other two groups. Besides, compared with presbycusis patients without cognitive decline, presbycusis patients with cognitive decline observed significantly increased nodal centralities in the frontal-prefrontal regions and decreased nodal centralities in the temporal-occipital regions. (3) Altered intra- and inter-modular connections were found mainly in the Frontal and Prefrontal-Subcortical modules. (4) The distribution of rich-club nodes in presbycusis patients changed, while no differences were found in the three connections among the three groups. All of these results not only helped to expand the understanding of the reorganization of the functional connections after presbycusis but also provided explicit evidence for disruption of brain functional network topology after cognitive decline.

In the framework of graph theory, there are three types of networks: regular, small-world, and random network, and these networks are judged by cluster coefficient and characteristic path length (Xia and Wang, 2010). Combining the advantages of regular and random networks, the small-world network has a higher clustering coefficient, which is favorable for local specialized processing, and a shorter characteristic path length, which is favorable for global distributed processing (Bullmore and Sporns, 2009; Xia and Wang, 2010; Bassett and Bullmore, 2017). Previous studies have shown that small-world characteristics exist in brain structures and functional networks, and changes in topology properties may lead to a variety of neuropsychiatric disorders such as Alzheimer's disease and Parkinson's disease (Rubinov and Sporns, 2010; Wang et al., 2013; Luo et al., 2015).

The increased cluster coefficient (Cp) and characteristic path length (Lp) can indicate the process of transferring networks from small-world networks to rule networks. The Cp reflects the level of local connectedness of a network (Lv et al., 2018), and the increased Cp indicates that the local brain network is enhanced, which are compensatory mechanisms that form clusters to preserve efficient communication. Wang et al. found that the normalized cluster coefficient and normalized characteristic path length are increased in mild cognitive impairment, and abnormality in these structural networks is associated with the slow speed of information processing (Wang et al., 2014). Both the Lp and the Eglob reflect the ability to communicate global information (Lv et al., 2018; Dai et al., 2019). The increased Lp and the decreased Eglob have been demonstrated to be associated with the decrease of transmission and integration function of long-distance brain interval information in type 2 diabetes and posttraumatic stress disorder (Lei et al., 2015; Xu et al., 2019). Consistently, Bai et al. and Wang et al. demonstrated that the increased Lp may have some correlations with poorer cognitive performance, corresponding to clinical manifestations such as cognitive, emotional, or language impairment following a hearing loss (Bai et al., 2012; Wang et al., 2013). The decreased sigma may reflect the imbalance in the differentiation and integration of brain networks in presbycusis patients without cognitive decline. All these changes in the global topological properties of these brain networks indicate that the neural network structure is abnormal in presbycusis patients. Similar findings can be found in other diseases, such as tinnitus (Lan et al., 2022), unilateral sudden sensorineural hearing loss (Xu et al., 2016), and lung cancer (You et al., 2021). Considering the previous studies, we speculated that the brain function of presbycusis patients may have been impaired in the early stage of hearing impairment. Under compensatory mechanisms, its local information processing capability is enhanced, consistent with the founding in type 2 diabetes (Xu et al., 2019). With the development of hearing impairment, the appearance of cognitive decline makes the impairment in brain function further aggravated. Compensatory mechanisms cannot compensate for the impairment in brain function, and its long-distance information transmission ability drops.

At the nodal level, compared with HCs, presbycusis patients had multiple nodes with abnormal betweenness centrality (Bc), degree centrality (Dc), and nodal efficiency (Ne), suggesting nodal reorganization in the global brain functional network. Bc and Dc measure the importance of a node in the network (Itahashi et al., 2014). Among these abnormal nodes, we noted that the nodes of the frontal lobe show both decreased Bc and Dc in presbycusis patients; considering the previous studies, these abnormal nodes may have reduced connectivity and efficiency for the communication with other regions (Sporns, 2011). However, compared with the HCs, the nodes showing both increased Bc and Dc in presbycusis patients were mainly in the precuneus, superior frontal gyrus, sup-occipital gyrus, and postcentral gyrus. Hence, we supposed that these nodes may reflect compensatory plasticity to keep efficient information communication across the functional network in presbycusis patients. In addition, compared with presbycusis patients without cognitive decline, presbycusis patients with cognitive decline showed some increased Bc and Dc nodes in the frontal lobe and some decreased nodes mainly in the occipital lobe and temporal lobe. The temporal lobe is responsible for processing auditory information and is also involved in cognitive functions such as memory and emotion (Yau et al., 2009; Hsu et al., 2012; Li et al., 2020); the occipital lobe dysfunction can lead to cognitive dysfunction that mainly manifests as memory deficits and motor perception disorders (Yeo et al., 2011; Wu et al., 2020). Nodal efficiency (Ne) is an index that evaluates the capacity of a node for information communication (Liao et al., 2017). All these abnormal nodes may have reduced connectivity and efficiency for communication with other regions. Considering the nodal local efficiency results, hearing impairment may disrupt the distributed networks of the frontal region, while cognitive decline may disrupt the distributed networks of the occipital and temporal regions. Besides, the increased nodal strength may reflect the compensatory process reaction of hearing impairment and cognitive decline. These findings accord with the change in global properties and might be attributed to compensatory efforts. When nerves are damaged, they may first activate the compensation mechanism and then gradually form a new neural network, resulting in functional replacement (Sun et al., 2016). Further efforts are needed to prove this hypothesis.

Modular architecture refers to the node group that is tightly connected within a local area but sparsely connected externally, enabling a balance between energy cost and communication efficiency (Andric and Hasson, 2015; Sporns and Betzel, 2016). Modular analysis showed that the intra-modular interaction of the frontal lobe module and the inter-modular interaction of the prefrontal-subcortical module increased in presbycusis patients with cognitive decline when compared with HCs. Besides, compared with presbycusis patients without cognitive decline, the inter-modular interaction of the prefrontal subcortical module increased in presbycusis patients with cognitive decline. Previous studies (Yau et al., 2009; Hsu et al., 2012) have shown that the frontal module is the main brain area involved in the cognitive impairment, which is associated with impairments in cognitive functions such as information processing speed, memory, and emotion. The prefrontal module is considered to be involved in the processing of visual, taste, smell, and somatosensory information (Rolls, 2004). Yuan et al. (2021) demonstrated the negative effects of chronic stress on prefrontal-subcortical functional connectivity in female squirrel monkeys. Changes in intra-modular and inter-modular connections in MELAS patients implied that there may be inappropriate integration between bottom-up sensory input and top-down regulation in MELAS patients (Wang et al., 2020). The high connectivity between the two modules can also be explained as a result of the compensation effect. These changes indicate that the balance between energy cost and communication efficiency has been broken in presbycusis and cognitive impairment.

In our study, all participants in the three groups had highly connected nodes, which were described as a rich-club organization (Sporns, 2011; Van Den Heuvel and Sporns, 2011). The distribution pattern of rich-club nodes had less difference among each group; it meant that the architecture of rich-club nodes was reorganized during the presbycusis stage. The same rich-club nodes in the three groups were mainly in the supplementary motor area, precuneus, and paracentral lobule. Compared with HCs, the newly formed rich-club nodes in presbycusis patients with cognitive decline were in the prefrontal module and temporal module, while the newly formed rich-club nodes in presbycusis patients without cognitive decline were mainly in the temporal and occipital module. In other words, for presbycusis patients with/without cognitive decline, the change of the nodes in the temporal module from non-rich club to rich club was a functional reorganization to compensate for the dysfunction of the nodes in the occipital module. Besides, some studies (Sporns, 2011; Van Den Heuvel and Sporns, 2011, 2012) suggested that the connections among rich-club nodes are central to the integration of information among different subsystems of the human brain. There were no significant differences in the rich-club connectivity, local connectivity, or feeder connectivity among the three groups. Given that the connectivity was not affected, we supposed that the brain network is thus compensated with the high efficiency after presbycusis and cognitive dysfunction.

The results of this study indicated that topological properties of brain functional connections are changed in presbycusis patients. In addition, the changes in presbycusis patients with cognitive decline were more significant than in those without cognitive decline. The alterations in presbycusis patients without cognitive decline suggested that brain networks have extensive plastic reorganization to produce functional replacement. However, with the development of hearing impairment, the cognitive decline appears to break the balance. When increased BC in the right postcentral gyrus (PoCG.R) and increased DC in the left superior occipital gyrus (SOG.L) are observed in patients with presbycusis, more interventions are needed to prevent further development of hearing impairment resulting in cognitive decline. These findings provided meaningful insights for further understanding the neural mechanism of presbycusis and cognitive decline.

The main limitations of this study include the following. (1) This research was a cross-sectional study with a relatively small sample size. Therefore, it is difficult to make a direct causal inference about the relationship between changes in topological properties and presbycusis patients with/without cognitive decline. Further longitudinal fMRI studies and larger samples are required to confirm the current findings. (2) We divided the whole brain into 90 regions using the AAL template to conduct the functional brain networks and omitted the cerebellum. There is no unified standard template for the division of brain regions in previous brain network research, while different segmentation schemes exhibit distinct topological structures. To avoid the influence of the varying node definitions of the different brain atlas templates, further studies are required to establish and use a unified standard template. (3) Although earplugs have been used to minimize the noise generated by MRI scans, we were unable to eliminate the possibility that noise might affect the results, which should be considered in future research.

## Conclusion

In this study, graph theory analysis based on rs-fMRI was used to study the topological properties of brain network in presbycusis patients with/without cognitive decline and found that both global and node properties were changed. In addition, the changes were more pronounced in presbycusis patients with cognitive decline than in presbycusis patients without cognitive decline. Particularly, abnormal changes in these properties in presbycusis patients may compensate for cognitive impairment by mobilizing additional neural resources, such as the increase of nodal centrality and efficiency of multiple brain regions.

## Data Availability Statement

The original contributions presented in the study are included in the article/supplementary material, further inquiries can be directed to the corresponding author/s.

## Ethics Statement

The studies involving human participants were reviewed and approved by Research Ethics Committee of Nanjing Medical University. The patients/participants provided their written informed consent to participate in this study.

## Author Contributions

BG, YX, and Y-CC designed the experiment, analyzed the data, and drafted the paper for the work. CX, LX, and SS helped to acquire the clinical and fMRI data. J-JX helped to revise the paper critically for important intellectual content. YW and QY did the financial support, review, and final approval of the paper to be published. All authors have read and approved the final manuscript.

## Funding

This study was supported by the 333 Project of Jiangsu Province (No. BRA2017154), the Natural Science Foundation of Jiangsu Province (No. BK20201220), and the Medical Science and Technology Development Foundation of Nanjing Department of Health (No. YKK21133).

## Conflict of Interest

The authors declare that the research was conducted in the absence of any commercial or financial relationships that could be construed as a potential conflict of interest.

## Publisher's Note

All claims expressed in this article are solely those of the authors and do not necessarily represent those of their affiliated organizations, or those of the publisher, the editors and the reviewers. Any product that may be evaluated in this article, or claim that may be made by its manufacturer, is not guaranteed or endorsed by the publisher.
